# Deciphering the Forebrain Disorder in a Chicken Model of Cerebral Hernia

**DOI:** 10.3390/genes11091008

**Published:** 2020-08-27

**Authors:** Yingfeng Tao, Xiaoliu Zhou, Xinting Zheng, Shijun Li, Chunyan Mou

**Affiliations:** Key Laboratory of Agricultural Animal Genetics, Breeding and Reproduction of Ministry of Education, College of Animal Science and Technology, HuaZhong Agricultural University, Wuhan 430070, China; yingfengtao@webmail.hzau.edu.cn (Y.T.); xiaoliuzhou@webmail.hzau.edu.cn (X.Z.); chen.li@webmail.hzau.edu.cn (X.Z.); lishijun@mail.hzau.edu.cn (S.L.)

**Keywords:** astrocyte, brain, cerebral hernia, chicken, DNA methylation, neuron, sphenoid bone, telencephalon

## Abstract

Cerebral hernia in crested chicken has been characterized as the protrusion of cerebral hemispheres into the unsealed skull for hundreds of years, since Charles Darwin. The development of deformed forebrain (telencephalon) of cerebral hernia remains largely unknown. Here, the unsealed frontal skull combined with misplaced sphenoid bone was observed and potentially associated with brain protuberance. The shifted pallidum, elongated hippocampus, expanded mesopallium and nidopallium, and reduced hyperpallium were observed in seven regions of the malformed telencephalon. The neurons were detected with nuclear pyknosis and decreased density. Astrocytes showed uneven distribution and disordered protuberances in hyperpallium and hippocampus. Transcriptome analyses of chicken telencephalon (cerebral hernia vs. control) revealed 547 differentially expressed genes (DEGs), mainly related to nervous system development, and immune system processes, including astrocyte marker gene *GFAP*, and neuron and astrocyte developmental gene *S100A6*. The upregulation of *GFAP* and *S100A6* genes in abnormal telencephalon was correlated with reduced DNA methylation levels in the promoter regions. The morphological, cellular, and molecular variations in the shape, regional specification, and cellular states of malformed telencephalon potentially participate in brain plasticity and previously reported behavior changes. Chickens with cerebral hernia might be an interesting and valuable disease model to further explore the recognition, diagnosis, and therapy of cerebral hernia development of crested chickens and other species.

## 1. Introduction

Cerebral hernia in chicken was observed and recorded as being strongly associated with feather crest in the 17th century. More recently, it was described that the cerebral hemispheres were squeezed into the membranous frontal bone region of the skull, leading to the malformation of the brain and cranium [1]. Further investigation revealed that the telencephalon and other brain tissues (the cerebellum and diencephalon) were misshaped with elongated telencephalon and misplaced from each other, exhibiting great differences from the closely packed compartments of wild type chicken brains [1,2]. The cerebral hernia phenotype was reported to be accompanied with a “Crest” with elongated feathers on top of the head [1,2,3] in Polish, Houdan, Paduan, and other chicken breeds [1,2]. Nonetheless, the correlation between cerebral hernia and feather crest phenotypes in chickens has been very controversial since the publication by Charles Darwin [4]. Whether the cerebral hernia and feather crest represent two independent phenotypes, or whether one is the prime cause and affects the other as a side effect is unclear. Some studies described a strong association of cerebral hernia with the chicken feather crest [1]. However, there is not a consensus on this due to limited knowledge. Eventually, two studies partially clarified the enigma by establishing chicken resource population and found out that the cerebral hernia and feather crest were potentially controlled by a single gene with autosomal recessive inheritance and autosomal incomplete dominant inheritance, respectively [1,5]. More solid data are needed to support this notion in the future.

Like other animals, the skull of the chicken protects the brain from external forces and restricts the brain in a relatively fixed position to permit proper function [6]. However, the frontal bone of chickens with cerebral hernia is partially membranous to form an irregular skull hole. Therefore, the telencephalon, which is partially free from the skull restriction, is pushed upward to form a protuberance that resists pressure poorly [1]. In addition, in the cerebral hernia of the duck, fat tissue invades the brain cavity, which may be the cause of the brain abnormalities [7,8]. Cerebral hernia in the crested chicken brought about many pathological peculiarities, such as excessive accumulation of cerebrospinal fluid (CSF) [1]. Importantly, in humans, hydrocephalus is a common neurosurgical disease caused by an imbalance between CSF secretion and reabsorption [9]. Chicken with cerebral hernia and hydrocephalus may be potentially useful disease model to further explore the human brain abnormalities.

The telencephalon of birds can be divided into seven regions including the hyperpallium (H), mesopallium (M), nidopallium (N), arcopallium (A), striatum (St), pallium (P), and hippocampus (Hp). These regions perform auditory, visual, somatic motor, and somatosensory functions [10,11]. The central nervous system consists mainly of neurons and glial cells. The function of neurons is to transmit information through synaptic structures [12]. The intelligence of animals and humans and their information processing capacities are closely related to the state of the neurons, including the number and density of neurons, and the inter-neuronal distance [13,14]. Birds like corvids and parrots have extremely high neuronal packing densities to process information, which partially explains their more advanced cognitive abilities compared to other birds [13,15]. Furthermore, glial cells are essential for nervous system development, neuronal generation, migration and plasticity, synaptogenesis, and homeostasis [12]. Astrocytes are one type of the glial cells connected with both the neurons and the blood vessels and essential to nourish the neurons [16], and to enhance neuronal plasticity and memory [17]. Glial fibrillary acidic protein (GFAP) is a major intermediate filament protein that functions as a marker for astrocytes. When the brain or central nervous system are injured, the expression of *GFAP* is increased to positively regulate astrogliosis and maintain homeostasis [12,18,19]. Besides, *S100A6* has been reported as a new marker of neural stem cells and astrocyte precursors in the adult mouse hippocampus [20]. In adult rats, *S100A6* is also expressed in subpopulations of neurons and glial cells of the nervous system [21]. *S100A6* may be involved in neuronal degeneration and regeneration in pathophysiological and traumatic brain injury rat [22,23]. DNA methylation is an epigenetic modification that plays a crucial role in coordinating the timing and magnitude of gene expression during brain development [24]. In mice, DNA methylation of the *GFAP* promoter region regulates the transition from neurogenesis to astrocyte development in the developing brain [25]. In human cancer, *S100A6* expression is upregulated by epigenetic regulation [23].

Previous experiments focused mainly on the anatomical and behavioral peculiarities of crested chickens with cerebral hernia, including general descriptions of the skulls and brains, and mental retardation [1,2]. However, why these birds are so different in brain phenotype in association with behavior abnormalities remains unknown. The molecular mechanisms and cellular changes involved in cerebral hernia remain unclear. A detailed investigation would be helpful to better understand the mystery of cerebral hernia in chickens. Chickens with cerebral hernia could serve as an experimental model to elucidate the histological and molecular differences of the malformed telencephalon, and provide theoretical bases and guidance to cerebral hernia development of chicken and other species.

## 2. Materials and Methods

### 2.1. Experimental Animals

The resource family of chickens was generated using an F2 design by crossing chickens with spontaneously mutated cerebral hernia with White Leghorn chickens to obtain F1 progeny. These chickens were then crossed to gain F2 progeny. Segregation of character occurred in the F2 generation, producing chickens with cerebral hernia phenotype (cerebral hernia type chickens) and normal phenotype chickens (wild type without cerebral hernia). The wild type and cerebral hernia type chickens were raised at the local experimental chicken farm (Wuhan, China). Six chickens derived from two groups (*n* = 3) were sacrificed using immersion in CO_2_ gas under euthanasia according to the standard procedure [26,27,28]. The brain (telencephalon) of each chicken was carefully removed and collected excluding the skulls and dura at 28 days after hatching (P28) and immediately placed in cold PBS. The brain was divided into two parts. The left part was triturated and placed in liquid nitrogen for RNA extraction. The right part was fixed in 4% paraformaldehyde at 4 °C for approximately one week. All the experiments on animals were approved by the Standing Committee of Hubei People’s Congress and the ethics committee of Huazhong Agricultural University.

### 2.2. Nissl Staining

Nissl staining was used to identify the functional regions of the telencephalon and explore the morphological differences and changes in the state and distribution of the neurons in the telencephalon between wild type and cerebral hernia type chickens as described previously [10,29]. Thionin (0.1 g) was added to 100 mL double distilled water to prepare Nissl staining solution. A series of telencephalon sagittal sections (5 μm thick) were prepared, dewaxed, and stained with thionin solution for 20 min, followed with 95% alcohol, xylene, and neutral gum to seal the stained sections. All stained sections were photographed using Olympus BX53 microscope (Tokyo, Japan).

Image-Pro-Plus 6.0 software (Media Cybernetics, Rockville, MD, USA) was used to analyze the brain parenchymal area, and to determine the number and density of neurons. Briefly, the images of comparable compartments of telencephalon sagittal sections were imported into Image-Pro-Plus 6.0 software. Each region of the telencephalon was manually selected to calculate the actual size and neuron number, respectively. The approximate density of neurons in each region of the telencephalon is figured out by using the total number of neurons in each region divided by the area of that region. Comparisons between the different groups including independent samples were statistically analyzed using Student’s *t*-tests (*n* = 3).

### 2.3. Hematoxylin and Eosin (H&E) Staining

H&E staining was used to determine the differences of histological features in the telencephalon between wild type and cerebral hernia type chickens. A series of fixed chicken brain samples were dehydrated using an alcohol gradient, processed in paraffin, and cut into 5 μm thick sagittal sections, following standard procedures. The sagittal sections were used for H&E staining and the stained sections were photographed to determine brain morphological and structural differences.

### 2.4. Methylation Template Purification and DNA Modification

Genomic DNA was obtained from the chicken brain (telencephalon) samples using a TIANamp Genomic DNA Kit (TIANGEN, Beijing, China). Genomic DNA (1 μg) was modified using an EpiTect^R^ Bisulfite Kit (QIAGEN, Hilden, Germany), according to the operating instructions.

### 2.5. PCR Amplification, Cloning, and Sequencing of Modified DNA

The online MethPrimer software (http://www.urogene.org/cgi-bin/methprimer/methprimer.cgi) was used for the prediction of CpG islands and primer design for Bisulfite sequencing PCR (BSP). The bisulfite-converted DNA was amplified in the *GFAP* promoter and exon1 regions and *S100A6* promoter region using touchdown PCR. The primers for PCR amplification are listed in Table S1. The reaction system included bisulfite-converted DNA, 10× EpiTaq PCR Buffer, MgCl_2_, dNTP, forward and reverse primer, EpiTaq HS, and water (TaKaRa, Kyoto, Japan). The PCRs were carried out using the following procedures. Initially, multiplex-touchdown PCR was performed, followed by one cycle of 98 °C for 10 s; with 0.5 °C decrements from 62 °C to 54.5 °C (*GFAP*) or 60 °C to 52.5 °C (*S100A6*) for 30 s and 72 °C for 40 s per cycle; then a further 20 cycles of 98 °C for 10 s, 54 °C (*GFAP*) or 52 °C (*S100A6*) for 30 s, and 72 °C for 40 s. The products of the PCR were purified using an Easy Pure Quick Gel Extraction Kit (TransGen, Beijing, China). The purified DNA fragments were linked using a pMD18-T Vector Cloning Kit (TaKaRa, Kyoto, Japan) and transformed into *Escherichia coli* DH5α-competent cells (Kangwei, Beijing, China) for further replication. The identified clones were selected for sequencing. Independent amplification experiments were performed for each treatment. The analysis of DNA methylation in the two groups (three independent samples per group) was derived from six independent biological samples (*n* = 3). Six positive clones were selected for sequencing analysis per sample (*n* = 6). A total of 18 positive clones were sequenced per group and applied for subsequent analysis. The methylation level of each sampled cytosine was estimated as the number of reads reporting a C, divided by the total number of reads reporting a C or T. Methylation profiles were drawn using CpGviewer software [30].

### 2.6. Illumina Sequencing and Data Analysis

RNAs were extracted from six independent chicken brain (telencephalon) samples from the two groups (*n* = 3) using TRIzol reagent and the RNA concentration was measured using a Qubit^®^ RNA Assay Kit in a Qubit^®^2.0 Flurometer (Life Technologies, Carlsbad, CA, USA). RNA integrity was assessed using the RNA Nano 6000 Assay Kit and the Bioanalyzer 2100 system (Agilent Technologies, Santa Clara, CA, USA). All samples had a RIN ≥ 7.9. The sequencing libraries were generated at Novogene (Beijing, China) using the NEBNext^®^ UltraTM RNA Library Prep Kit for Illumina^®^ (NEB, Ipswich, MA, USA) following the manufacturer’s recommendations, and the library quality was assessed on the Agilent Bioanalyzer 2100 system. After cluster generation, the libraries were sequenced on an Illumina Hiseq platform, with 150 bp paired-end reads. The clean reads were generated and aligned to the reference chicken genome (gallus gallus 6.0 ftp://ftp.ensembl.org/pub/release96/fasta/gallus_gallus/dna/Gallus_gallus.GRCg6a.dna.toplevel.fa.gz) using Hisat2 v2.0.5 [31], and the number of reads mapped to each gene was counted by the feature Counts v1.5.0-p3. The fragments per kilobase of exon model per million reads mapped (FPKM) of each gene was calculated to obtain the expression level based on the length of the gene and the read count mapped to it. For biological replicates, genes with |fold change| > 1.5 and *p* < 0.05 were regarded as differentially expressed genes (DEGs) (Table S2).

### 2.7. Data Deposition

The sequencing data from this study is available in the database of National Center for Biotechnology Information (NCBI) Sequence Read Archive (SRA) under the accession number PRJNA647159.

### 2.8. GO, KEGG Enrichment, and PPI Analysis of DEGs

The clusterProfiler R package was used to analyze Gene Ontology (GO) enrichment of DEGs. GO terms with *p* < 0.05 were considered significantly enriched by DEGs. Kyoto Encyclopedia of Genes and Genomes (KEGG) enrichment analyses of DEGs were implemented by clusterProfiler R package. Protein–protein interaction (PPI) based on DEGs was analyzed by the STRING database (https://string-db.org/). Cytoscape software (version 3.7.1, https://cytoscape.org/) was used to visualize the PPI network.

### 2.9. Quantitative Real-Time PCR (qRT-PCR) Validation

The expression level of selected differentially expressed mRNAs were validated by qRT-PCR. *Glyceraldehyde 3-phosphate dehydrogenase (GAPDH)* was used as an internal reference gene. The primers for qRT-PCR are shown in Table S3. The qRT-PCR was performed on a Roche LightCycler^R^ 96 using iTaq^TM^ Universal SYBR^R^ Green Supermix (Bio-Rad, Hercules, CA, USA). The amplification protocol comprised one cycle at 95 °C for 5 min, followed by 45 cycles of 95 °C for 15 s and 60 °C for 1 min. The relative gene expression was calculated by the 2^−∆∆Ct^ method with average cycle thresholds. Comparisons between the different groups from six independent samples from each group were statistically analyzed using the Student’s *t*-test (*n* = 6).

### 2.10. Immunohistochemistry

Immunohistochemistry was used to detect markers in chicken brain (telencephalon) tissue. The telencephalon samples were dehydrated using an ethanol gradient series. Next, the samples were embedded in paraffin and cut into 5 μm thick sections. The telencephalon sections were dewaxed for antigen retrieval and incubated with primary antibodies GFAP (1:200, Sigma-Aldrich, St. Louis, MO, USA, G3893), NeuN (1:400, Millipore, Burlington, MA, USA, MAB377), and MAP2 (1:400, Millipore, Burlington, MA, USA, AB5622) at 4 °C for 18 h. The sections were further incubated with a secondary antibody from an immunological kit (Proteintech, Wuhan, China) for 1 h at 37 °C, and then stained with DAB staining (1:50) for visualization, followed with hematoxylin counter-staining. All immunohistochemical experiments were repeated at least three times.

### 2.11. Statistical Analyses

All the data are presented as mean ± SEM. All statistical tests were performed using GraphPad Prism software version 6 software. Student’s *t*-test was used to assess the differences between two groups. Statistically significant differences were determined at *p* < 0.05.

## 3. Results 

### 3.1. Morphological Characterization of Cerebral Hernia in Crested Chickens

**Figure 1:**
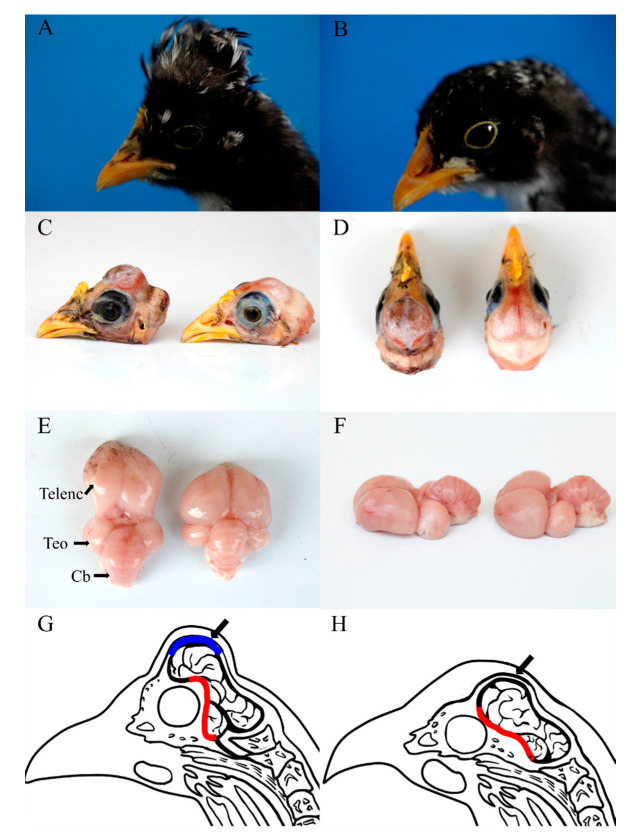
Anatomy and morphology of cerebral hernia type and wild type chickens. (**A**,**B**) The appearance of chickens with cerebral hernia (cerebral hernia type) (**A**) and without cerebral hernia (wild type) (**B**). (**C**,**D**) The differences of frontal bone between cerebral hernia type and wild type at P28 are shown in the lateral view (**C**) and dorsal view (**D**), respectively (left, cerebral hernia type; right, wild type). (**E**,**F**) The external morphology of brain in cerebral hernia type and wild type chickens at P28 from the dorsal (**E**) and lateral view (**F**), respectively (left, cerebral hernia type; right, wild type). (**G**) The diagram revealed abnormal development of the sphenoid bone and membranous frontal bone in the cerebral hernia type chickens (misplaced sphenoid bone is denoted in red, and the membranous frontal bone is denoted in blue region and with a black arrow). (**H**) The diagram showed the morphology of the sphenoid bone and frontal bone in wild type chickens (sphenoid bone is denoted in red, and frontal bone is indicated by a black arrow). Cb, cerebellum; Telenc, telencephalon; TeO, optic tectum.

There were significant differences between the phenotypes of chickens with or without cerebral hernia. In chickens with cerebral hernia, the feathers on top of the head were elongated to form a high and upright crest (Figure 1A), compared with the short head feathers with no crest in wild type chickens (Figure 1B). Underneath the feather crest, the partial frontal skull was transformed from bone to membranous lining that covered the telencephalon leading to the formation of an irregular skull hole (Figure 1C,D). The telencephalon was not restricted by the continuous skull bone, and protruded from the skull hole to form a protuberance termed the cerebral hernia. CSF was generally observed in association with the cerebral hernia after removal of the membranous cover (Supplementary Figure S1A,B). Close inspection revealed that the telencephalon and other regions (the cerebellum and diencephalon) of the chicken brain with cerebral hernia were loosely united and separated from each other, rather than being closely packed together in wild type chicken brain (Figure 1E,F).

The location of the sphenoid bone that is positioned beneath and supports the telencephalon changed from the moderate angle to the horizon in wild type chickens to a nearly vertical angle in chickens with cerebral hernia. The alteration of sphenoid bone lifted the upper telencephalon that was positioned at an angle of approximately 80° to the horizon in cerebral hernia type chickens, compared with that of approximately 50° in wild type chickens. Hence, this abnormity of the sphenoid bone was possibly the major cause of the distinctive external brain morphology of chickens with cerebral hernia. A diagram of the chicken head was drawn to demonstrate the anatomical differences between the two phenotypes. The vertically located sphenoid bone (red region) and membranous frontal lining (blue region) were distinct in the head of cerebral hernia type chickens (Figure 1G), compared with those of moderately angled sphenoid bone and continuously sealed frontal bone (red region and black arrow) in the wild type chickens (Figure 1H). The birds with cerebral hernia are also observed to be generally quiet or inactive which is consistent with previous reports. Overall, the huge differences in the skull, sphenoid bone, behavior, and brain—especially the telencephalon—were observed between the cerebral hernia and wild type chickens.

### 3.2. Telencephalon Deformation Accompanied with Ectopic Neurons

**Figure 2:**
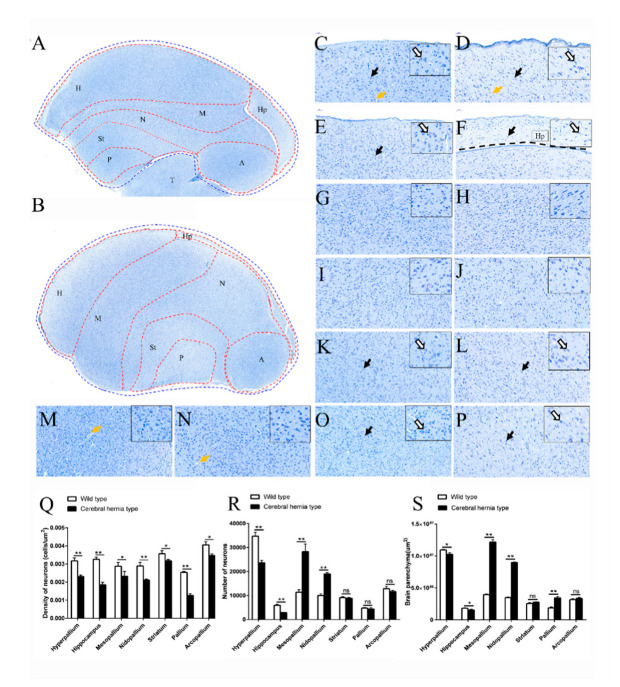
Comparison of the telencephalon in cerebral hernia type and wild type chickens using Nissl stain. (**A**,**B**) The telencephalon encircled by the blue dotted line is divided into seven regions by marking the boundaries for each region with a red dotted line in wild type chickens (**A**) and cerebral hernia type chickens (**B**). Scale bars = 500 µm. (**C**,**D**) The sagittal sections of telencephalon in H region are presented in wild type chickens (**C**) and cerebral hernia type chickens (**D**). (**E**,**F**) The sagittal sections of telencephalon in Hp region are presented in wild type chickens (**E**) and cerebral hernia type chickens (**F**). (**G**,**H**) The sagittal sections of telencephalon in M region are presented in wild type chickens (**G**) and cerebral hernia type chickens (**H**). (**I**,**J**) The sagittal sections of telencephalon in N region are presented in wild type chickens (**I**) and cerebral hernia type chickens (**J**). (**K**,**L**) The sagittal sections of telencephalon in St region are presented in wild type chickens (**K**) and cerebral hernia type chickens (**L**). (**M**,**N**) The sagittal sections of telencephalon in A region are presented in wild type chickens (**M**) and cerebral hernia type chickens (**N**). (**O**,**P**) The sagittal sections of telencephalon in P region are presented in wild type chickens (**O**) and cerebral hernia type chickens (**P**). The neurons are marked with black arrows and open arrows indicating lower and higher magnification, respectively in (**C**–**P**). The blood vessels are indicated with yellow arrows in (**C**–**P**). The Hp region in cerebral hernia type chickens is located above the black dotted line (**F**). Scale bars = 50 µm. (**Q**) Quantitative analysis of the density of neurons in the telencephalon showed the decreased tendency in all seven regions in cerebral hernia type chickens. (**R**) The number of neurons in the telencephalon is increased in M and N, but decreased in H and Hp. (**S**) Quantitative analysis of the brain parenchyma areas demonstrating enlargement of M, N, and P regions, and reduced size of H and Hp regions in cerebral hernia vs. wild type chickens. H, hyperpallium; Hp, hippocampus; M, mesopallium; N, nidopallium; St, striatum; A, arcopallium; P, pallium. Data are presented as mean SEM (from three independent samples per group). (*n* = 3); * *p*< 0.05, ** *p*< 0.01 (Student’s *t*-test).

In chickens with cerebral hernia, the telencephalon was greatly altered in morphology with elongation and protuberance (Figure 1E). No studies have documented aberrant functional regions of the telencephalon, except for a general morphological description. Nissl staining was used to detect the Nissl bodies inside the neurons in the telencephalon sagittal sections. The telencephalon could be divided into seven regions (H, Hp, M, N, St, P, and A) in the wild type (Figure 2A) and cerebral hernia type chickens (Figure 2B) with relative differences in position and shape of each region.

**Figure 3:**
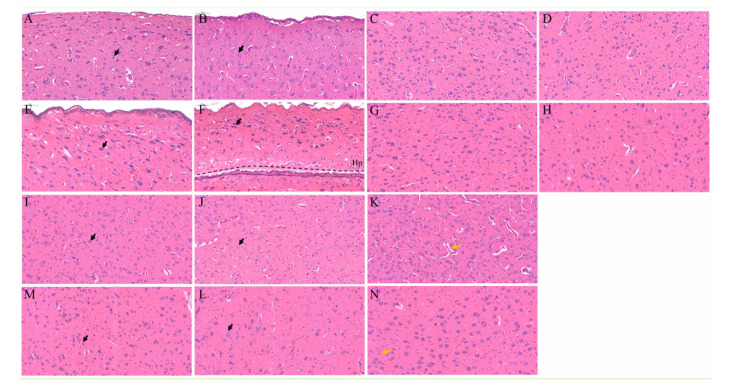
Identification of histological features of telencephalon in cerebral hernia and wild type chickens based on hematoxylin and eosin staining. (**A**–**N**) The H, Hp, M, N, St, P, and A regions are represented in figure (**A**,**E**), (**B**,**F**), (**C**,**G**), (**D**,**H**), (**I**,**M**), (**J**,**L**), and (**K**,**N**) in wild type and cerebral hernia type chickens, respectively. The state of cells and blood vessels are marked with black and yellow arrows, respectively. The Hp region in cerebral hernia type is located above the black dotted line in image F. Scale bars = 50 µm.

It was striking that the location of the M, N, St, and P regions in the telencephalon were greatly changed in cerebral hernia type chickens compared with that of wild type chickens. The P region displayed a marked change in position, nearly taking over the space previously occupied by T region (Figure 2A,B). Almost every region in the telencephalon of cerebral hernia type chickens was detected with the variation of shape, and the Hp region was greatly altered with elongated length and narrowed width (Figure 2A,B), followed by moderate changes of the H, M, N, St, and P, and with a subtle variance of the A region. Further inspection showed the decreased areas of the H and Hp regions and the increased areas of the M, N, and P regions in the telencephalon of cerebral hernia type chickens (Figure 2S). Nissl staining also revealed that the number of neurons was significantly decreased in H and Hp regions and increased in the M and N regions, respectively (Figure 2R). The density of neurons was decreased in each region of telencephalon in cerebral hernia type chickens, with the greatest reduction in the H, Hp, and P regions (Figure 2Q). Similar findings were apparent in H&E stained samples (Figure 3E,F,L).

Importantly, the neurons in the telencephalon of wild type chickens were regularly arranged with clear nucleolus and obvious Nissl bodies in the cytoplasm (Figure 2C,E,G,I,K,M,O). H&E staining further demonstrated the histological features and cell-state changes of each region in the telencephalon, which was consistent with the results of Nissl staining (Figure 3A–D,I–K). However, in chickens with cerebral hernia, the neurons in the H, Hp, M, St, and P regions showed smaller neuron body and nucleus pyknosis (Figure 2D,F,H,L,P), while those in the N and A regions had no obvious changes (Figure 2J,N). H&E staining also revealed that the nucleolus of neurons was weakly basophilic in each region and approximately hypertrophic in the P region (Figure 3L) of the telencephalon in the cerebral hernia type chickens (Figure 3E–H,M). In addition, fewer blood vessels were observed in telencephalon of cerebral hernia type chickens compared with rich vessels in those of wild type chickens (Figure 2D,F,N and Figure 3E,F,N). The collective analyses showed that the number and density of neurons and the size and shape of the specified regions in telencephalon were significantly changed in chickens with cerebral hernia.

### 3.3. Functional Annotation of DEGs in Deformed Telencephalon in Chickens with Cerebral Hernia

**Figure 4:**
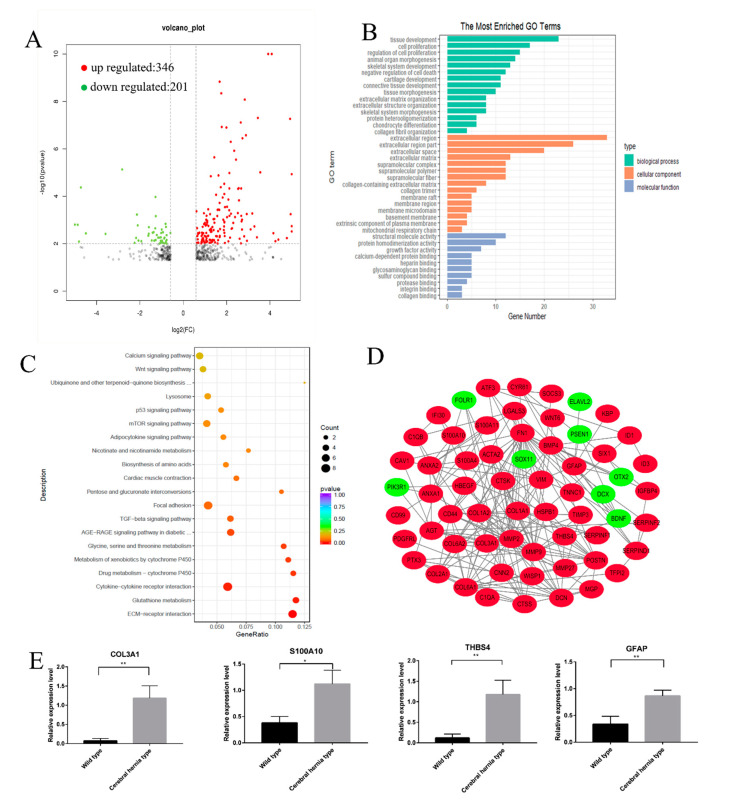
Transcriptomic annotation of telencephalon derived from chicken with or without cerebral hernia. (**A**) The differentially expressed transcripts in the telencephalon of wild type and cerebral hernia type chickens are grouped into upregulated and downregulated transcripts, which are marked with red and green dots, respectively. The criteria set up for the enrichment are |fold change| > 1.5 and *p* < 0.05. (**B**) The top 30 enriched GO terms for the differentially expressed transcripts in the telencephalon between wild type and cerebral hernia type chickens are listed as biological process (green), cellular components (yellow), and molecular function (blue). The criterion set up for the enrichment is *p* < 0.05. (**C**) The top 20 enriched KEGG pathways for the differentially expressed transcripts are listed and presented as the number of genes (indicated by the spot size) with significance (indicated *p*-value of the pathway with the spot color). (**D**) An mRNA–mRNA interaction network is constructed using the potential candidate genes involved in brain, nervous system, and blood–brain barrier (BBB) development. The upregulated and downregulated genes are denoted in red and green, respectively. (**E**) Expressions of randomly selected DEGs involved in brain and nervous system development are validated by qRT-PCR. The gene expression trends of *COL3A1*, *S100A10*, *THBS4*, and *GFAP* are consistent with the tendency of RNA sequencing results. The qRT-PCR data are presented from six independent samples per group as mean SEM (*n* = 6); * *p* < 0.05, ** *p* < 0.01 (Student’s *t*-test).

RNA sequencing was performed to investigate the molecular network regulating the arrangement of the telencephalon of cerebral hernia type chickens compared with wild type chickens (Figure 1E). Illumina sequencing of six cDNA libraries derived from the telencephalon tissue samples generated a total of 66,960,579 and 69,760,620 raw reads for Wild type and Cerebral hernia type samples, respectively. (Table S4). The sequencing result revealed 547 DEGs, which included 346 upregulated and 201 downregulated genes derived from two groups (three independent samples for each group) telencephalon transcriptomes (cerebral hernia vs. without cerebral hernia) (Figure 4A). The DEGs were further analyzed for GO terms and KEGG enrichment. Further analysis showed that the GO terms of DEGs were highly enriched in nervous system development and immune system processes (Figure 4B and Supplementary Figure S2). There were five GO terms that were highly correlated with nervous system development, including neuron apoptotic processes, neuron death, positive regulation of neuron differentiation, neuron projection, and dendritic tree. GO terms enriched in the immune system included regulation of immune system process, leukocyte mediated immunity, immune response regulating signaling pathway, activation of immune response, and T cell proliferation (Table 1). KEGG analyses enriched several important pathways related to brain development, such as the ECM-receptor interaction, TGF-β, p53, calcium, and mTOR signaling pathways (Figure 4C). It is interesting that the p53 signaling pathway was previously reported to regulate cell cycle, apoptosis, senescence, and metabolism, and accelerated DNA repair [32]. The result was consistent with the enrichment of the GO terms in aspects of the nervous system and immune system (Figure 4C).

The DEGs that potentially regulated brain development, nervous system, and blood–brain barrier were grouped and used to construct an mRNA-mRNA interaction network. A number of genes were associated with regulating brain cell matrix development (*COL3A1*, *COL1A1*, *COL1A2*, *COL6A1*, *COL6A2*, and *COL2A1*), neuroprotective action (*S100A11*, *S100A4*, *ANAX1*, *ANAX2*, *MGP*, *ATF3*, *ID1*, *HSBP1*, and *WISP1*), glial cells development (*GFAP*, *VIM*, *THBS4*, *S100A10*, *HBEGF*, and *LGALS3*), and blood–brain barrier (*TIMP3*, *CD99*, *MMP2*, *MMP9*, *MMP27*) (Figure 4D). A handful of genes (*COL3A1, S100A10, THBS4, GFAP*) that were potentially involved in brain and nervous system development were selected to validate the gene expression by qRT-PCR (Figure 4E). The upregulation of four selected genes in the telencephalon of cerebral hernia type chickens compared with wild type chickens as revealed by qRT-PCR, was consistent with those of the RNA sequencing. Together, these results demonstrate that there are large variations in the complex network regulating the morphological differences of the telencephalon in the cerebral hernia phenotypes.

### 3.4. Inspection of Neurons in Chicken Telencephalon with or without Cerebral Hernia by Immunohistochemistry

**Figure 5:**
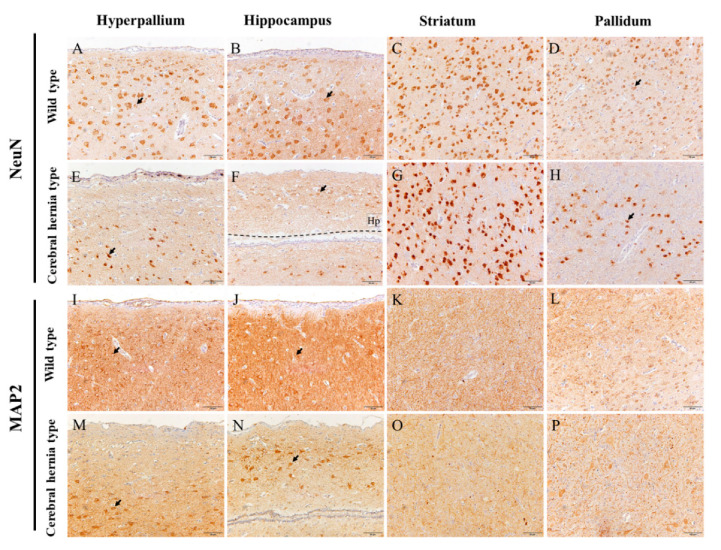
Abnormal neurons and dendrites are identified with NeuN and MAP2 antibodies by using immunohistochemistry in the telencephalon of chickens with and without cerebral hernia. (**A**–**H**) The positive signals of NeuN are detected in the neurons of H, Hp, St, and P regions of the telencephalon of wild type (**A**–**D**) and cerebral hernia type (**E**–**H**) chickens, respectively. In the telencephalon of chickens with cerebral hernia, the number of neurons is sparse with shrinking of the neuron body and nucleus pyknosis in H and Hp regions (**E**,**F**). The neurons in the St and P regions had intensely stained signals (**G**,**H**). The Hp region in cerebral hernia is located above the black dotted line in image F. (**I**–**P**) The positive signals of MAP2 are identified in the H, Hp, St, and P regions of the telencephalon of wild type (**I**–**L**) and cerebral hernia (**M**–**P**) chickens, respectively. The dendrites are well developed in wild type chickens, while in cerebral hernia chickens, the dendrites are atrophied in the H, Hp, St, and P regions. The neurons and blood vessels are indicated with black and yellow arrows, respectively. Scale bars = 50 µm (all panels).

Nissl and H&E staining results indicated the abnormal neurons in the H, Hp, St, and P regions of the telencephalon. To explore these differences more accurately, we verified the mature neurons and dendrites by detecting the marker genes NeuN and MAP2, respectively, using immunohistochemistry. In the wild type chicken telencephalon, the neurons were clearly arranged and strongly labeled in the H and Hp regions with even distribution and clear nucleus (Figure 5A,B). Moreover, the labeled dendrites and the unlabeled nucleus were clearly visible, forming clear boundaries between them (Figure 5I,J). However, the neurons were obviously different in the H and Hp regions of telencephalon in chickens with cerebral hernia accompanied with moderately intense NeuN labeling with smaller neuron bodies, condensed nucleus with significantly reduced number and density, particularly on the edge of these regions (Figure 5E,F). The neuron dendrites were sparsely branched with light MAP2 labeling, and reduced MAP2 immunoreactivity, and lack of terminal formations (Figure 5M,N). In the St region, compared with wild type chickens, the neurons were intensely stained (Figure 5C,G), while the dendrites were weakly stained with unclear boundaries of nuclei (Figure 5K,O). In addition, in the P region of the telencephalon, NeuN staining indicated decreased neurons with relatively darker stains in the cerebral hernia type chickens compared with the wild type chickens (Figure 5D,H). Similarly, the greatest changes of dendrites were deformation and disorganization states in chickens with cerebral hernia (Figure 5L,P). These results suggest that the neurons in the telencephalon of chickens with cerebral hernia are abnormal and in an injured state, which may be one explanation for the abnormal behavior of these chickens.

### 3.5. Detection of Astrocytes in Chicken Telencephalon with or without Cerebral Hernia by Immunohistochemistry

**Figure 6:**
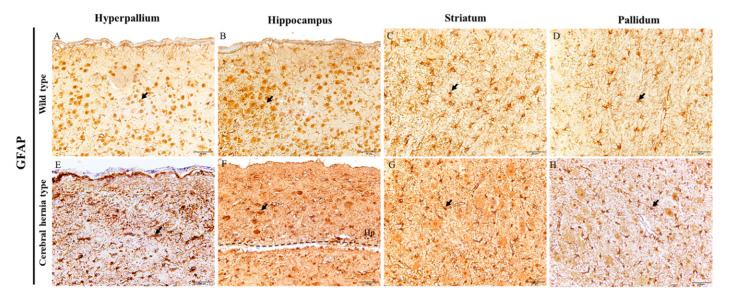
Immunohistochemistry of GFAP indicates abnormal astrocytes in telencephalon of chickens with cerebral hernia. (**A**–**D**) The astrocytes detected with GFAP antibodies are present with clear protuberances and even distribution in the H, Hp, St, and P regions of wild type chicken telencephalon, respectively. (**E**–**H**) The GFAP positive signals are localized in astrocytes in the H, Hp, St, and P regions of telencephalon in cerebral hernia type chickens, respectively. (**E**) The astrocytes are aggregated at some point in the H region. (**F**) The astrocytes are irregularly distributed in the Hp region. (**G**) Compared to wild type chickens, the astrocytes had intensely stained signals in the St region. (**H**) The protuberances of astrocytes are shrunken and blurred in the P region. Scale bars = 50 µm (all panels).

The results of RNA sequencing showed that the *GFAP* gene was differentially expressed in cerebral hernia type chickens compared with wild type chickens. Glial fibrillary acidic protein (GFAP) is used for the identification of astrocytes. In the H and Hp regions in wild type chickens, astrocytes can be observed with clear protuberances and an even distribution (Figure 6A,B), while that of cerebral hernia type chickens were aggregated, unevenly distributed with disordered protuberances, and differed in size (Figure 6E,F). In the St region, the astrocytes were intensely stained in the telencephalon of cerebral hernia type chickens (Figure 6C,G), which was consistent with the neuron staining results. In addition, in the P region of the wild type chickens, the protuberances of astrocytes were evenly distributed with a neat arrangement (Figure 6D). However, in chickens with cerebral hernia, the most obvious difference was that the labeled protuberances were smaller and blurrier than those in the control chickens (Figure 6H). These data indicate that the astrocytes in chickens with cerebral hernia are disordered and unevenly distributed. 

### 3.6. Evaluation of DNA Methylation of GFAP and S100A6 Genes in the Telencephalon of Chickens with or without Cerebral Hernia

**Figure 7:**
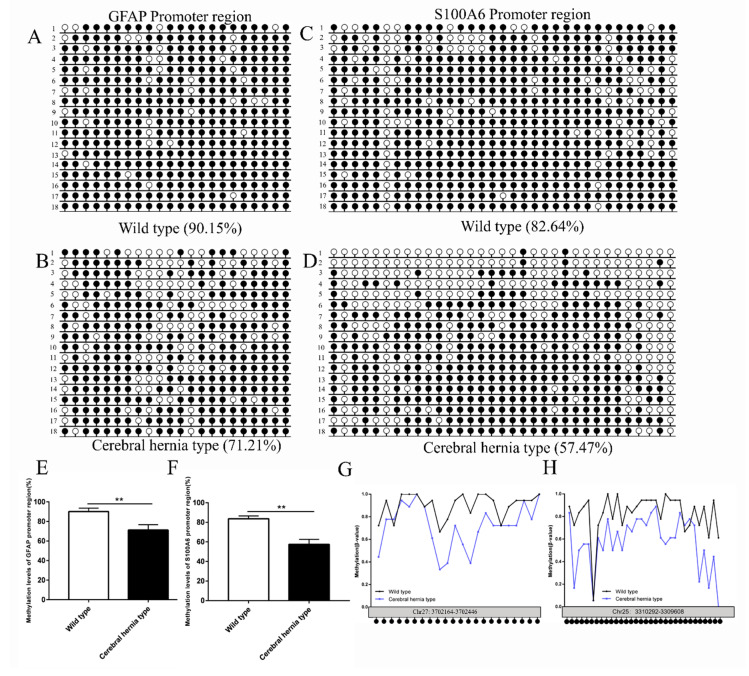
Methylation levels of *GFAP* and *S100A6* in promoter regions are decreased in telencephalon of cerebral hernia type chickens compared with wild type chickens. (**A**,**B**) Cytosine methylation profiles of *GFAP* promoter sequences in telencephalon of wild type (A) and cerebral hernia type (B) chickens, respectively. The cytosine methylation profiles were detailed as wild type 1 (rows 1, 2, 3, 4, 12, and 13), wild type 2 (rows 5, 6, 7, 9, 10, and 14), wild type 3 (rows 8, 11, 15, 16, 17, and 18), Cerebral hernia type 1 (rows 2, 3, 4, 5, 7, and 13), Cerebral hernia type 2 (rows 6, 8, 10, 11, 14, and 17) and Cerebral hernia type 3 (rows 1, 9, 12, 15, 16, and 18). (**C**,**D**) Cytosine methylation profiles of *S100A6* promoter sequences in telencephalon of wild type (**C**) and cerebral hernia type (**D**) chickens. The cytosine methylation profiles were detailed as wild type 1 (rows 1, 2, 4, 5, 12, and 14), wild type 2 (rows 3, 6, 8, 9, 13, and 17), wild type 3 (rows 7, 10, 11, 15, 16, and 18), Cerebral hernia type 1 (rows 1, 3, 7, 11, 14, and 15), Cerebral hernia type 2 (rows 2, 6, 8, 9, 13, and 16) and Cerebral hernia type 3 (rows 4, 5, 10, 12, 17, and 18). (**E**,**F**) Quantitative analysis results for methylation of *GFAP* and *S100A6* promoter sequences between wild type (**E**) and cerebral hernia type (**F**) chicken telencephalon, respectively. The methylation levels are both decreased in the *GFAP* and *S100A6* promoter sequences in telencephalon of chickens with cerebral hernia. (**G**,**H**) The methylation levels of *GFAP* (**G**) and *S100A6* (**H**) promoter regions at each CpG sites are shown by polyline diagram between wild-type and cerebral hernia-type chickens. For the above results, T vector cloning is obtained from three repeated samples, and six positive clones are selected for each biological sample (18 positive clones from three independent samples for each group) to calculate the percentage of methylation. The 18 methylation maps of each group are shown in A, B, C, and D.

In this study, the RNA sequencing analyses detected the upregulation of *GFAP* and *S100A6* genes in the telencephalon of chickens with cerebral hernia. A previous study on mice suggested that *GFAP* methylation status was tightly correlated with astrocyte differentiation [25]. In addition, *S100A6* is strongly expressed in hippocampal astrocytes and affects the proliferation and differentiation of neurons either directly or indirectly [23,33]. To identify whether DNA methylation regulates the expression of *GFAP* and *S100A6*, the DNA methylation patterns of *GFAP* and *S100A6* were detected in the telencephalon of wild type and cerebral hernia type chickens. Bisulfite sequencing PCR (BSP) was used to analyze the methylation status of CpG sites in the chicken *GFAP* and *S100A6* genes. A total of 22 CpG sites were amplified in the promoter region of *GFAP* gene (Figure 7A,B). In addition, 47 CpG sites were amplified in the exon1 region (Supplementary Figure S3A,B). Compared with the wild type, the sequencing data showed a lower DNA methylation level in both the promoter and exon1 regions of *GFAP* gene in cerebral hernia type chickens. In the promoter region, the average percentage of *GFAP* methylation in the wild type and the cerebral hernia type was 90.15% and 71.21%, respectively (Figure 7A,B). The methylation levels at each CpG site were further illustrated using a polyline diagram (Figure 7G). In the exon 1 region, prevalence of *GFAP* methylation in the wild type and the cerebral hernia type chickens was 92.91% and 80.97%, respectively (Supplementary Figure S3A,B). The methylation levels at each CpG site are shown in Supplementary Figure S3D. The quantitative analysis of *GFAP* methylation showed significant differences in both the promoter region and exon1 region between the wild type and cerebral hernia type chickens (Figure 7E and Supplementary Figure S3C).

In addition, BSP was used to analyze the methylation status of CpG sites in the chicken *S100A6* promoter region. A total of 33 CpG sites were amplified. The sequencing data showed that average percentage of DNA methylation levels in the promoter region of *S100A6* gene in the wild type and cerebral hernia type were 82.64% and 57.47%, respectively (Figure 7C,D), which demonstrated significant differences in the promoter region between the wild type and cerebral hernia type chickens (Figure 7F). The methylation levels at each CpG site of *S100A6* gene were illustrated using a polyline diagram (Figure 7H). The findings indicate that DNA methylation may regulate the expression of *GFAP* and *S100A6* genes to exert an important effect on functions of astrocytes and neurons, either directly or indirectly.

## 4. Discussion

### 4.1. Chickens with Cerebral Hernia Showed Behavior Abnormalities

Over a century ago, Darwin [4] and many other scholars extensively studied the interesting characteristics of cerebral hernias. A typical cerebral hernia can be observed in a number of birds including Polish, Houdan, and Paduan chickens and some crested ducks [1,2,3]. It is worth noting that the abnormal development of cerebral hernia leads to pre- and post-natal mortalities. Moreover, the membranous skull is prone to mechanical pressure and injuries which lead to unconsciousness. This produces huge economic losses in the poultry industry [8,34].

Darwin [4] reported that crested chicken strains were extremely stupid and seemed to suffer from mental retardation by showing that even when they are not far from their feeding ground, they could not find their way back [2]. In the 20th century, studies also reported that crested chickens showed poor learning ability, sluggish behavior, and often lacked interest in external events [2]. Furthermore, the crested ducks with cerebral hernia are found to go deaf and blind on one side during the third and fourth year of breeding, respectively [35]. This phenomenon is fully reflected in our research. Specifically, compared to wild type chickens, cerebral hernia type chickens displayed slower actions, delayed responses, reduced auditory acuteness, and inactivity. In addition, their vision is possibly impaired since they are unable to pay attention to approaching external objects. These results suggest that cerebral hernia may cause brain functional abnormalities resulting in various deficiencies in central nervous and behavioral abnormalities.

### 4.2. Cerebral Hernia Phenotype in Chickens Is Partially Caused by the Misplaced Sphenoid Bone

Interestingly, cerebral hernia is highly associated with chicken feather crest. Chickens with cerebral hernia invariably had feather crest, while chickens with feather crest did not always develop cerebral hernia. This triggered long debate regarding whether the cerebral hernia and the crest phenotypes are determined by one or two independent causative genes. The feather crest is potentially regulated by the *HOXC8* gene [5], while the cause of cerebral hernia has not been reported until now, except for a general morphological description. Why the brain is squeezed out of shape to form a cerebral hernia is unclear. There are several contradictory explanations. A report suggests that the cerebral hernia and crest phenotypes are monitored by two independent genes [36], whereas another study indicates the pleiotropic effects of one gene on both cerebral hernia and crest phenotypes [3].

Importantly, in this study, we provide an interesting finding and possible explanation of the brain deformation via anatomical observations, indicating that the changed position of the sphenoid bone is associated with the development of cerebral hernia. The further identification of correlation between sphenoid bone and deformed telencephalon would be interesting in the future. In humans, the sphenoid bone has many important functions. For example, the anterior part of the base of the cranial cavity is composed of the sphenoid bone, which helps to connect the neurocranium to the facial skeleton [37]. In addition, several important nerves including the optic nerve, oculomotor nerve, and trochlear nerve pass through the foramina and fissures of the sphenoid bone [37,38]. Sphenoid bone injury may cause vision loss or eye damage, such as symptoms of hemotympanum and cranial nerve palsies [37,39]. This may explain why chickens with cerebral hernia have poor vision and pay less attention to external objects. Importantly, sphenoid bone dysplasia leads to neurofibromatosis, which is characterized as the herniation of meningeal and cerebral structures, which triggers proptosis and facial disfiguration, and accompanied with the accumulation of CSF [40]. Consistent with this observation, we observed the protuberance of frontal bone and a generally excessive accumulation of CSF in cerebral hernia type chickens. The misplacement of sphenoid bone observed suggests that the changed sphenoid bone is potentially correlated with the abnormalities of telencephalon, as well as the injury caused by cerebral hernia.

### 4.3. Functional Impact of Abnormalities in Structure and Cell State of Telencephalon

Brain injury can induce neuronal death and abnormalities, in which misplaced neurons may make incorrect connections near inappropriate targets, leading to cognitive impairment, motor impairment, and frequent epilepsy, which increases the risk of Alzheimer’s disease (AD) and Parkinson’s disease, and the death rate in children and adults [41,42,43]. The development of cerebral hernia significantly alters the shape of the entire brain and displays the most significant changes in four regions—the H, Hp, St, and P—which may lead to brain injury. A mature neuron marker (NeuN), a dendrite marker (MAP2), and an astrocyte marker (GFAP), are used to further detect the abnormity of neurons and astrocytes.

We found that the density of neurons in the H, Hp, M, N, St, P, and A regions are all decreased in cerebral hernia type chickens, and the number of neurons in the H and Hp regions are also significantly lower. These observations indicated reduced connectivity among the neurons and decreased conduction speeds to partially explain why chickens with cerebral hernia are clumsy, unresponsive, and less intelligent. In addition, the abnormal shape and disordered distribution of the astrocytes further confirmed the abnormality in each region, especially in the H and Hp regions. This may disturb neuronal generation and migration, and the connections between neurons. Importantly, the auditory functional regions are mainly the N region, the ventral M region, adjacent parts of the caudal St region, and part of the A region, which assemble into a functional circuit [10]. Previous studies found that convergent neural circuits for vocal learning are accompanied by convergent molecular changes of multiple genes [44]. The state of the neurons in these regions significantly changed with nucleus pyknosis, decreased Nissl body numbers in the cytoplasm, and most importantly, the decreased density of neurons in N region, which can lead to hearing impairment. The visual regions consist of the ventral M near the entopallium, the adjacent N near the entopallium, and the adjacent St ventral to the entopallium and the H region. The motor and somatosensory functional regions are located in the anterior ventral M region and the adjacent anterior N region [10,45]. Similar phenomena have also been observed in these regions. In the telencephalon of cerebral hernia type chickens, not only the number of neurons decreased, but also the labeled nucleus of cells were smaller than those of wild type chickens. At the same time, the dendrites were underdeveloped and disordered, suggesting a negative effect on the eyesight, motor and somatosensory abilities in chickens with cerebral hernia. In addition, the Hp region that mainly controls memory is greatly deformed, elongated, and narrowed with decreased neurons and abnormal state, which may lead to amnesia in chickens with cerebral hernia. All the results suggest that the brain is injured with dysfunction in chickens with cerebral hernia.

### 4.4. Functional Analysis of DEGs in the Telencephalon of Chickens with or without Cerebral Hernia

The RNA sequencing data shows enrichment of two genes (*PSEN1* and *GRIA1*), which are related to learning or memory. In humans, *GRIA1* is involved in memory formation and schizophrenia pathology [46,47]. The expression of *PSEN1* is related to AD, which is a neurodegenerative disorder [48,49]. *PSEN1* mutation leads to a severe phenotype in AD astrocytes. The astrocytes play a key role in AD clearance while neurons are generally the main AD producers [49]. Importantly, *GFAP* gene expression can be significantly upregulated in AD, which is consistent with the upregulated *GFAP* expression in chickens with cerebral hernia. Interestingly, the *VIM* gene, which is a marker of immature astrocyte, is also upregulated in the telencephalon of cerebral hernia type chickens. We conclude that high *GFAP* expression is triggered to protect against brain deformation in cerebral hernia. Furthermore, several classical signaling pathways highly associated with brain development were also enriched, including the WNT, VEGF, and calcium (Ca^2+^) signaling pathways. Of these, Ca^2+^ regulation is partially controlled by *PSEN1* and is important in the progression of AD [50]. In addition, astrocytes can change the Ca^2+^ signaling activity of neurons, further demonstrating the importance of astrocyte-neuron interactions in AD [49]. The presently observed enrichment of the Ca^2+^ signaling pathway suggests that Ca^2+^ signaling may be involved in the development of cerebral hernia. Furthermore, the enrichment of genes related to the blood–brain barrier, including *CD99*, *MMP2*, and *MMP9* [51,52,53] indicates the potential damage of the blood–brain barrier during the development of cerebral hernia.

DNA methylation is related to neurodegenerative disorders such as AD, Parkinson’s disease, ataxia-related disorders, and spinocerebellar ataxias [24]. This leads to important regulation during the development of the nervous system. Interestingly, we found a strong correlation of *GFAP* expression with different degrees of methylation in the promoter and exon1 regions of the *GFAP* gene in cerebral hernia type chickens. *GFAP* is expressed in astrocytes, which are closely related to neuronal generation, migration, axon specification, and growth through circuit assembly and synaptogenesis [12,54]. Therefore, the results suggest that *GFAP* methylation plays an important role in regulating the abnormality of neurons in cerebral hernia. In addition, *S100A6* gene is crucial in cell proliferation and differentiation, calcium homeostasis, and neuronal degeneration during brain development [23]. In some tumors, *S100A6* expression has been negatively correlated with promoter methylation status [55]. The methylation level of *S100A6* is also lower in the promoter region and negatively correlated with the upregulated expression in the telencephalon of cerebral hernia type chickens. These results suggest the potential involvement of *S100A6* methylation in the regulation of developing abnormal telencephalon in cerebral hernia type chickens. In addition, the enriched other genes in the S100 family including *S100A1*, *S100A4*, *S100A10*, and *S100A11* were closely related to brain development and all upregulated in the telencephalon of chickens with cerebral hernia. *S100A1* is mainly involved in the regulation of Ca^2+^ signals and can cause pathology in AD with its overexpression in the brain [56]. *S100A4* is involved with the growth factor family receptor ErbB4 and promotes neuronal survival [57]. *S100A10* is widely distributed in vertebrates in regions including the brain, and plays a key role in membrane transport, vesicle secretion, and endocytosis [58]. *S100A11* can protect against neuronal cell apoptosis [59]. The expressions of *S100A1*, *S100A4*, *S100A10*, and *S100A11* are regulated by DNA methylation in medulloblastoma [60] and may have potential impacts on the disorder of telencephalon in cerebral hernia. The collective results indicate that epigenetics plays an important role in the development of cerebral hernia.

## 5. Conclusions

This study provides anatomical and morphological descriptions that reveal the fundamental differences in each region of the brain in chickens with or without cerebral hernia. In the telencephalon of chickens with cerebral hernia, the P region is misplaced and the Hp region is elongated and narrowed. Moreover, the state of neurons and astrocytes is ectopic in almost every region. Importantly, we provide new evidence to propose that the misplaced sphenoid bone with a steeper angle than that of the wild type is correlated with the development of cerebral hernia. In addition, the enriched genes associated with the development of nervous and immune systems underlie the occurrence of abnormal telencephalon of cerebral hernia. The upregulation of *GFAP* and *S100A6* genes is highly correlated with the reduced levels of DNA methylation in the promoter region that regulate the proliferation, differentiation, and migration of astrocytes and neurons in the telencephalon of chickens with cerebral hernia, and eventually lead to the mental retardation and abnormal behavior. This is the first report elucidating the anatomical, morphological alterations, and global gene expression changes in deformed telencephalon of cerebral hernia. Chicken with cerebral hernia may be an additional disease model to facilitate the understanding of brain dysplasia as well as the behavior changes associated with it and eventually provide meaningful insights into cerebral hernia development in chicken and other species.

## Figures and Tables

**Table 1:** List of enriched GO terms involved in nervous and immune system development

Description	Genes
Neuron apoptotic process	**PSEN1** CEBPB **BDNF**
Neuron death	**PSEN1** CEBPB **BDNF**
Positive regulation of neuron differentiation	**PSEN1 BDNF**
Neuron projection	**PSEN1 BDNF NFIB** RHOC **GABRQ** PTGS2
Dendritic tree	**PSEN1** BDNF
Regulation of immune system process	CEBPB **CD28 PSEN1** ANXA1 CAV1
Leukocyte mediated immunity	**CD28**
Immune response-regulating signaling pathway	**CD28 PSEN1**
Activation of immune response	**CD28 PSEN1**
T cell proliferation	**CD28** CEBPB ANXA1

The bold font represents the downregulated DEGs.

## Supplementary Materials

The following are available online at https://www.mdpi.com/2073-4425/11/9/1008/s1, Figure S1. Morphological characterization after removal of the membranous frontal skull in chickens with and without cerebral hernia; Figure S2. Enrichment of GO terms for downregulated and upregulated genes; Figure S3. Methylation levels of GFAP in the exon1 region are decreased in telencephalon of chickens with cerebral hernia; Table S1. Primers used for the touchdown PCR; Table S2. Differentially expressed transcripts in the telencephalon between wild type and cerebral hernia type chickens; Table S3. Primers used for qRT-PCR; Table S4. Statistics of Raw reads and clean reads in the telencephalon of wild type and cerebral hernia type chickens.
