# Supplementary material for: Deciphering the Forebrain Disorder in a Chicken Model of Cerebral Hernia
Source: Genes (Basel). 2020 Aug 27;11(9):1008. doi: 10.3390/genes11091008 (PMC7564858; doi:10.3390/genes11091008)
Supplement: Supplementary file 1 [file genes-11-01008-s001.zip › supplementary materials/Supplementary figure legends.docx]

**
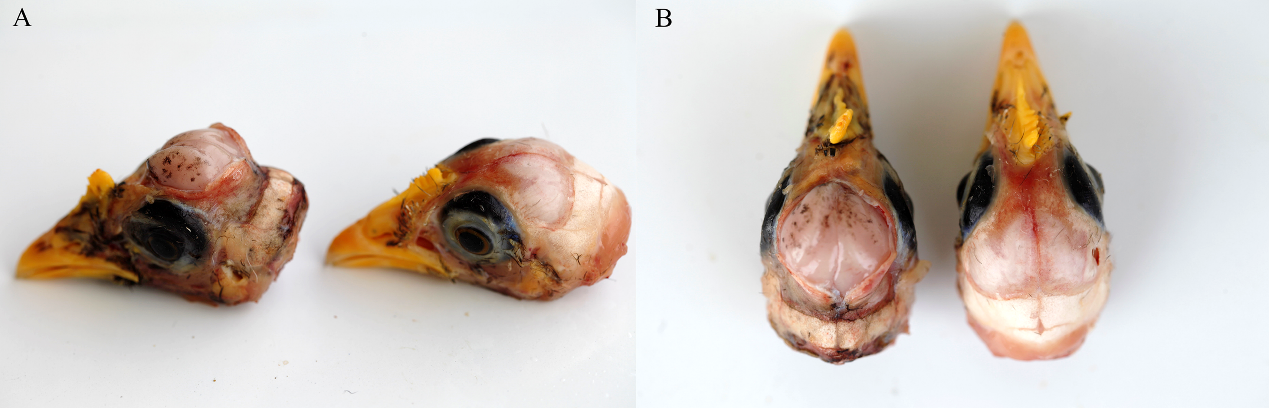
**

**Figure S1. Morphological characterization after removal of the membranous frontal skull in chickens with and without cerebral hernia.** (A-B) The phenotypic differences between the cerebral hernia type after removal of the membranous frontal bone and the wild type at P28, from lateral (A) and dorsal view (B), respectively (left, cerebral hernia type; right, wild type).


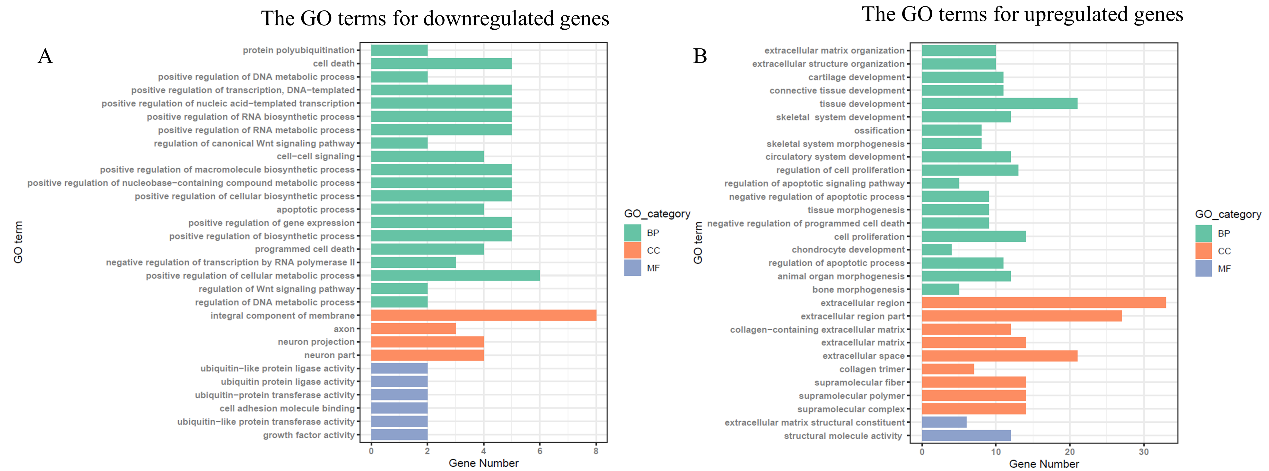


**Figure S2. Enrichment of GO terms for downregulated and upregulated genes.**

The enriched GO terms for the downregulated expressed transcripts (A) and upregulated expressed transcripts (B) in the cerebral hernia type chickens compared with the wild type chickens are listed as biological process (green), cellular components (yellow), and molecular function (blue). The criterion set up for the enrichment is P < 0.05.


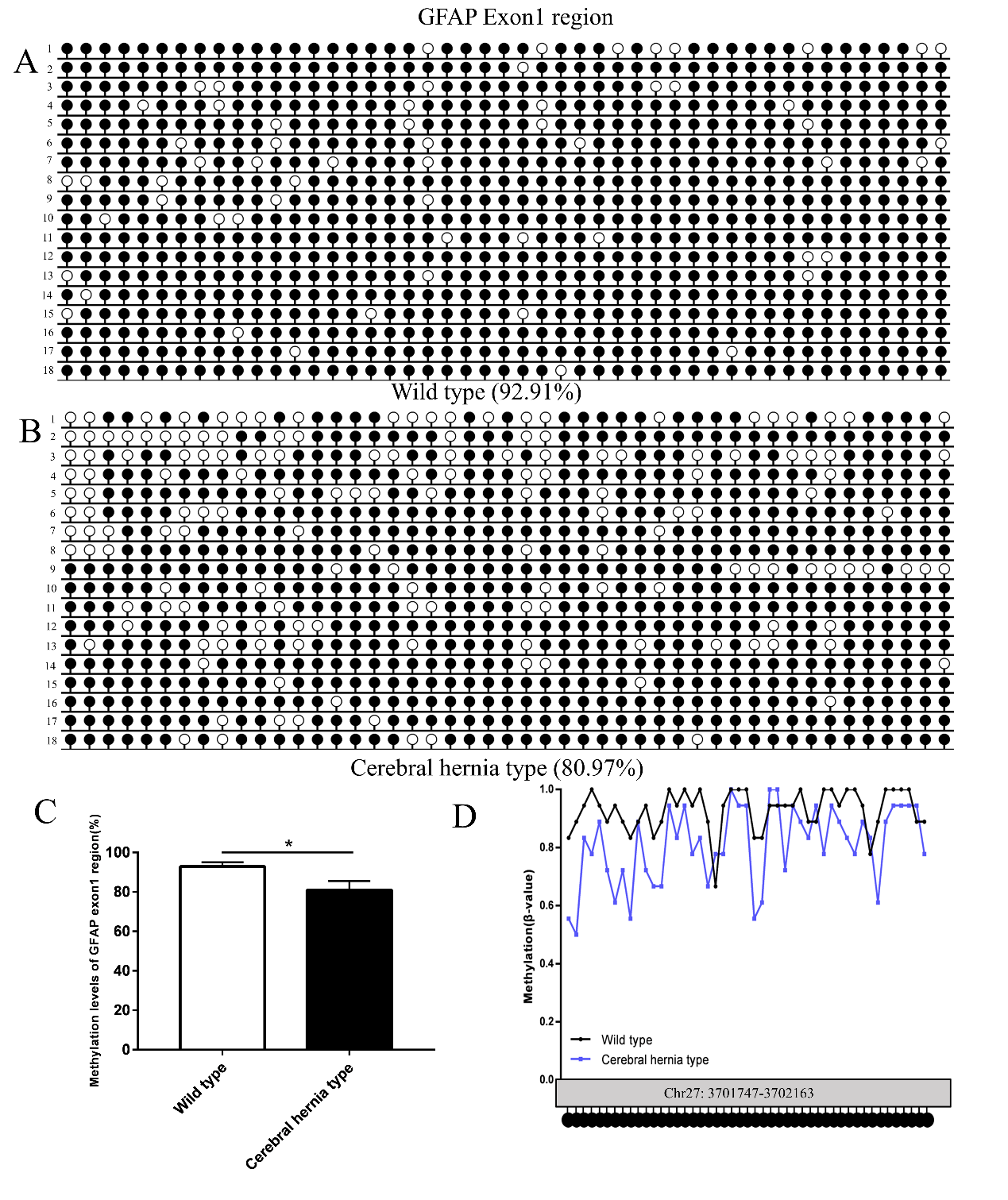


**Figure S3. Methylation levels of *GFAP* in the exon1 region are decreased in telencephalon of chickens with cerebral hernia.** (A-B) Cytosine methylation profiles of *GFAP* exon1 are listed in telencephalon of in wild-type (A) and cerebral hernia type (B) chickens, respectively. The cytosine methylation profiles were detailed as wild type 1 (rows 3, 5, 7, 9, 10 and 11), wild type 2 (rows 4, 12, 13, 14, 15 and 17), wild type 3 (rows 1, 2, 6, 8, 16 and 18), Cerebral hernia type 1 (rows 1, 4, 7, 8, 9 and 10), Cerebral hernia type 2 (rows 11, 13, 14, 15, 16 and 18) and Cerebral hernia type 3 (rows 2, 3, 5, 6, 12 and 17). (C) Quantitative analysis results of the methylation of *GFAP* exon1 sequences between wild type and cerebral hernia type chicken telencephalon. The methylation level is significantly lower in *GFAP* exon1 sequences in telencephalon of chickens with cerebral hernia compared to wild type chickens. (D) Polyline diagram of the methylation levels of *GFAP* exon1 at each CpG site between wild type and cerebral hernia type chickens. Data are presented from three independent samples with six positive clones per sample to calculate the percentage of methylation using mean SEM (n= 3); *P < 0.05, **P < 0.01 (Student’s t-test).

**Supplementary Table S2** Differentially expressed transcripts in the telencephalon between wild type and cerebral hernia type chickens.
