# Supplementary material for: Deciphering the Forebrain Disorder in a Chicken Model of Cerebral Hernia
Source: Genes (Basel). 2020 Aug 27;11(9):1008. doi: 10.3390/genes11091008 (PMC7564858; doi:10.3390/genes11091008)
Supplement: Supplementary file 1 [file genes-11-01008-s001.zip › supplementary materials/Supplementary table1íótable3 and table4.docx]

Table S1. Primers used for the touchdown PCR

| Genes | Forward (5'–3') | Reverse (5'–3') |
| --- | --- | --- |
| GFAP | TTCGGGGTGTATTTTATAGTG | CAACTCCTCAACCAAACC |
| S100A6 | TAGTTTTATTAGCGTGAGTG | AAAATTACTCACCATCCCC |

Table S3. Primers used for qRT-PCR.

| Genes | Forward (5'–3') | Reverse (5'–3') |
| --- | --- | --- |
| COL3A1 | CCCAGGTCTTCCAGGAATGAG | CATTACACCTGGCTGACCACG |
| S100A10 | GTGCGCTGATGGAGAAGGAGT | CTGGAAGCCCACTTTGCCATC |
| THBS4 | GCAGTAGAGTTACGCACCTTC | CAGTGTACTGAGGAGCAGATTC |
| GFAP | GACCAGCCTGGACACCAAATC | CTACACCACCTCCTTGTGCTCC |

Table S4. Statistics of Raw reads and clean reads in the telencephalon of wild type and cerebral hernia type chickens

| Sample | Raw reads | Clean reads | Raw Base(G) | Clean bases(G) | Mapping ratio |
| --- | --- | --- | --- | --- | --- |
| Wild type 1 | 21,248,643 | 20,533,368 | 6.37 | 6.16 | 87.92% |
| Wild type 2 | 24,495,115 | 23,554,087 | 7.35 | 7.07 | 86.71% |
| Wild type 3 | 21,216,821 | 20,572,069 | 6.37 | 6.17 | 86.92% |
| Cerebral hernia type 1 | 22,091,595 | 21,554,322 | 6.63 | 6.47 | 87.29% |
| Cerebral hernia type 2 | 22,274,746 | 21,641,767 | 6.68 | 6.49 | 86.88% |
| Cerebral hernia type 3 | 25,394,279 | 24,862,228 | 7.62 | 7.46 | 86.47% |
